# PET imaging of the autonomic myocardial function: methods and interpretation

**DOI:** 10.1007/s40336-015-0139-6

**Published:** 2015-09-10

**Authors:** Walter Noordzij, Riemer H. J. A. Slart

**Affiliations:** Department of Nuclear Medicine and Molecular Imaging, University of Groningen, University Medical Center Groningen, Hanzeplein 1, P.O. Box 30001, 9700 RB Groningen, The Netherlands

**Keywords:** PET, Myocardial innervation, Methods, Interpretation

## Abstract

Cardiac positron emission tomography (PET) is mainly applied in myocardial perfusion and viability detection. Noninvasive imaging of myocardial innervation using PET is a valuable additional methodology in cardiac imaging. Novel methods and different PET ligands have been developed to measure presynaptic and postsynaptic function of the cardiac neuronal system. Obtained PET data can be analysed quantitatively or interpreted qualitatively. Thus far, PET is not a widely used clinical application in autonomic heart imaging; however, due to its technical advantages, the excellent properties of the imaging agents, and the availability of tools for quantification, it deserves a better position in the clinic. From a historical point of view, the focus of PET software packages for image analysis was mainly oncology and neurology driven. Actually, commercially available software for cardiac PET image analysis is still only available for the quantification of myocardial blood flow. Thus far, no commercial software package is available for the interpretation and quantification of PET innervation scans. However, image data quantification and analysis of kinetic data can be performed using adjusted generic tools. This paper gives an overview of different neuronal PET ligands, interpretation and quantification of acquired PET data.

## Introduction

Positron emission tomography (PET) is successfully used in cardiac applications since the 1980s and showed the potential of this technique for the characterisation of myocardial perfusion and metabolism. In the beginning as a research tool, but later PET has been developed as a clinical imaging tool.

PET scanning has also been established for cardiac autonomic innervation assessment, mainly in the research setting. It holds true that PET conveys higher methodological demands as well as less general availability. Nevertheless, high spatial and temporal resolution together with sequential attenuation correction considerably improves the quality of the scans and, therefore, resulting in higher accuracy as compared with SPECT [1]. Furthermore, PET enables regional absolute quantification from dynamic data [2]. There is also increasing need for non-invasive identification of new and refinement of existing methods of risk stratification to particularly identify patients at risk for ventricular tachyarrhythmia’s and cardiac death. The presence of disrupted cardiac sympathetic innervation is a predictor of future sudden cardiac death after myocardial infarction [3]. PET, being the most optimal technique for imaging sympathetic innervation, can be applied to identify patients at high risk of fatal arrhythmias [4]. At present, an implantable cardioverter defibrillator (ICD) is the most effective treatment option to prevent death from ventricular arrhythmia, and it is superior compared to the use of anti-arrhythmic drugs alone [5–7]. However, not all patients eventually suffer from ventricular arrhythmia. Actually, the minority of patients (approximately 30 %) benefit from prophylactic ICD treatment. There is a clinical need for better identification of those patients at risk for life-threatening arrhythmia and thus successful ICD therapy.

The available PET radiotracers enable the evaluation of both the presynaptic and postsynaptic parts of the myocardial autonomic innervation. Taking this in account, PET offers an attractive methodology for innervation assessment. This paper gives an overview of different neuronal PET ligands, interpretation and quantification of acquired PET data.

## PET ligands to imaging the autonomic myocardial function

### Presynaptic sympathetic innervation

A number of radiotracers have been developed, evaluated and deployed targeting presynaptic neuronal function (uptake-1 norepinephrine reuptake pathway), postsynaptic α- and β-adrenoceptor density, and second messenger systems (adenylate cyclase/cyclic AMP and phospholipase C/inositol trisphosphate cascades). While the majority of clinical applications to date have utilised analogues of norepinephrine including iodine-123 labelled metaiodobenzylguanidine ([^123^I]-MIBG) with SPECT, the most commonly used PET tracer for imaging of presynaptic sympathetic function is carbon-11 labelled meta-hydroxyephinephrine ([^11^C]-mHED), which is also a norepinephrine analogue. In contrast to the other PET tracers [^11^C]-epinephrine and [^11^C]-phenylephrine, [^11^C]-mHED is not susceptible to breakdown by monoamine oxidase and catechol-*O*-methyltransferase. For [^11^C]-mHED, this does not result in radiolabelled metabolites and, therefore, no need for metabolite correction, which is necessary for [^11^C]-epinephrine and [^11^C]-phenylephrine [8, 9].

**Fig. 1:**
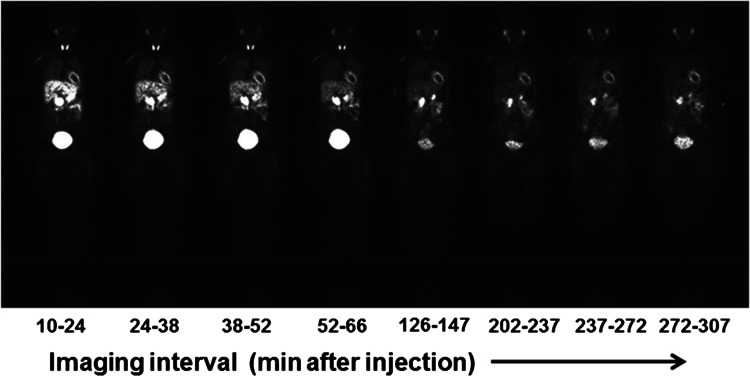
Representative sequence of whole-body LMI1195 coronal images at mid-myocardial level in human volunteer. Each whole-body image is scaled to maximum value within that image. This research was originally published in JNM. Sinusas AJ, Lazewatsky J, Brunetti J, Heller G, Srivastava A, Liu YH, Sparks R, Puretskiy A, Lin SF, Crane P, Carson RE, Lee LV. Biodistribution and radiation dosimetry of LMI1195: first-in-human study of a novel 18F-labeled tracer for imaging myocardial innervation. J Nucl Med. 2014;55:1445–1451. © by the Society of Nuclear Medicine and Molecular Imaging, Inc

More recently, a PET analogue of MIBG has been developed and analysed, attempting to capitalise on the clinical experience with the iodinated SPECT analogue and the higher spatial resolution of PET. The compound, N-[3-bromo-4-(3-[^18^F]-fluoro-propoxy)-benzyl]-guanidine ([^18^F] LMI1195) which can be easily labelled by direct [^18^F]-fluorination of a brosylate precursor, has undergone preclinical testing, in which cardiac uptake was well defined compared to liver. Imaging studies in rabbits with desipramine blockade or 6-hydroxydopamine denervation demonstrated a correlation between [^18^F]-LMI1195 retention and uptake-1 density without a corresponding difference in myocardial blood flow for either model [10]. Recently, a human safety, whole-organ biodistribution, and radiation dosimetry of [^18^F]-LMI1195 (Fig. 1) were evaluated in a phase 1 clinical trial [11]. These preliminary data suggest that [^18^F] LMI1195 is well tolerated and yields a radiation dose comparable to that of other commonly used PET radiopharmaceuticals.

### Postsynaptic sympathetic innervation

In addition to presynaptic sympathetic innervation imaging using norepinephrine analogues, it is also possible to measure postsynaptic β-adrenoceptor density on the cardiomyocyte. Changes in β-adrenoceptor density are of importance in the development of heart failure: β-adrenoceptor density is downregulated as a consequence of enhanced sympathetic drive in heart failure [12, 13]. However, the clinical use of these receptor–ligands has been limited to only a few studies and still faces significant challenges. At present, [^11^C]-CGP-12177 is the most widely used PET tracer for imaging β-adrenoceptor density [14, 15]. This tracer is suggested feasible for clinical utility due to high receptor affinity and fast plasma clearance. However, its synthesis is rather complicated. The isopropyl analogue of [^11^C]-CGP-12177 S-4-(3-([^11^C]-isopropylamino)-2-hydroxypropoxy)-2H-benzimidazol-2-one ([^11^C]-CGP12388) can be labelled easier and has a similar biodistribution and retention as [^11^C]-CGP-12177 [16, 17]. [^11^C]-CGP-12388 has the potential to monitor β-adrenoceptor remodelling induced by therapeutic regimen. However, this has not yet been visualized. Recent developments have led to the introduction of [^18^F] labelled β_1_-adrenocepter antagonist ICI89406 ([^18^F]-FICI); unfortunately, this PET compound proofed to be aspecific [18]. The frequency of β-blocker therapy in heart failure population limits the utility of adrenoceptor radioligands, as accurate quantitative imaging necessitates discontinuation of this therapy. As such, there has been exploration of the potential to image signal transduction following adrenergic receptor stimulation. While few of these compounds have yet been evaluated in a clinical setting, the preclinical evidence supports long-term development of multitracer studies, including candidate radiotracers of intracellular signalling, for instance [^11^C]-rolipram [19–21].

### Parasympathetic innervation

The parasympathetic nervous system is the autonomic counterpart of the sympathetic or adrenergic nervous system, and consists of a dense network of nerves with widespread distribution of muscarinic and nicotinic receptors, which mediate the response to released acetylcholine. At present, two PET tracers have been clinically validated for invasive imaging of parasympathetic innervation. These tracers are the highly specific muscarinic receptor antagonist [^11^C]-MQNB and the nicotinic agonist 2-[^18^F]-F-A-85380 [22, 23]. A derivate of the selective antagonist of vesicular acetylcholine transporters has only been used in a preclinical setting [24]. Very recently, some exploratory experience was gained on the visualisation of nicotinic acetylcholine receptors in the vascular wall, which are also stimulated by exogenous nicotine [25, 26]. Additional studies are needed to assess its value in parasympathetic innervation imaging.

## PET data analysis and interpretation

### [^11^C]-mHED

In short, acquisition of data occurs after injection of approximately 350 MBq (range 200–400 MBq) of [^11^C]-mHED (Fig. 3). During dynamic imaging for 60 min, heart rate and blood pressure are monitored continuously. Since retention of [^11^C]-mHED is dependent on myocardial perfusion, a myocardial perfusion PET should always be performed before the [^11^C]-mHED acquisition. Acquired data are then corrected for attenuation and for residual perfusion PET tracer activity.

Originally, kinetic analysis of [^11^C]-mHED data was performed according to compartment models, which require good understanding of the different compartments to which the tracer is distributed. Different compartments are connected by rate constants, which describe the exchange of tracer between them, using blood sampling (arterial preferable) as input and reference. The different equations, measured blood and tissue activity curves, and consequent estimated rate constant, make compartment models complex and susceptible to noise, As an alternative to these complex procedures, [^11^C]-mHED uptake is now commonly quantified through a retention index, which is defined as the ratio of the activity in the myocardium in the final image of a 40- or 60-min dynamic sequence to the integral of the image-derived arterial blood time–activity curve. A volume of interest (VOI) for the input function is placed in the basal plane; the VOI for tissue curves is place within the left ventricle wall. Acquisition of [^11^C]-mHED data can also be performed using ECG gating, for simultaneous analysis of LV volumes and contractility [27].

Since the myocardial uptake of [^11^C]-mHED is relatively constant during the first 8 h after administration, this retention index is very reliable. However, the blood pool integral increases over time due to circulating metabolites. The contribution of metabolites can be suppressed using a correction method, when acquiring data at later time points that 40- or 60-min post-injection [28]. Recent studies found a close correlation between [^11^C]-mHED retention index and late [^123^I-MIBG] heart-to-mediastinum rate [29].

**Fig. 2:**
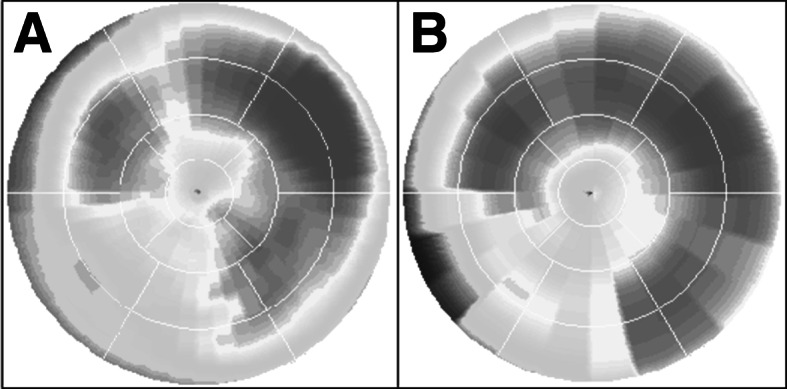
An example of a matched perfusion and innervation defect in the inferoseptal wall of the left ventricle. **a** Polar map of rest nitrogen-13 labelled ammonia PET, indicating myocardial infarction in the inferoseptal wall, **b** polar map of [^11^C]-mHED uptake in the same patient, with a defect in the same area as the myocardial infarction

**Fig. 3:**
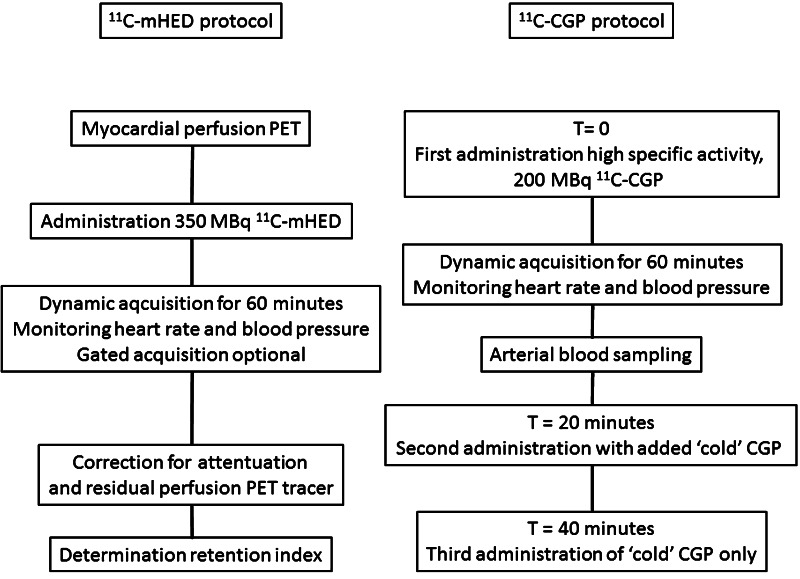
Flow charts showing the summary of the acquisition protocols of **[** ^11^C**]**-mHED and **[** ^11^C**]-**CGP

Distribution of [^11^C]-mHED throughout left ventricular myocardium in healthy normal individuals is regionally homogeneous with high uptake in all myocardial segments [30]. Therefore, the PET tracer [^11^C]-mHED is an attractive non-invasive method to quantify the activity and distribution of sympathetic innervation. Visual interpretation of bull’s eye plots, analogue to and alongside those of myocardial perfusion, provides information for global innervation status. However, when using the same 17-segment model as in perfusion imaging analysis, detailed information about perfusion–innervation relationships can be obtained (Fig. 2). Mean [^11^C]-mHED retention can be determined for those areas with both normal perfusion (>80 % of the maximum myocardial blood flow) and innervation (>75 % of the segment with maximum [^11^C]-mHED retention), areas with a mismatch pattern: normal perfusion and decreased cardiac sympathetic innervation (<75 % of the segment with maximum [^11^C]-mHED retention), and both abnormal perfusion (<80 % of the maximum myocardial blood flow) and innervation (Fig. 3).

### [^11^C]-CGP quantification

**Fig. 4:**
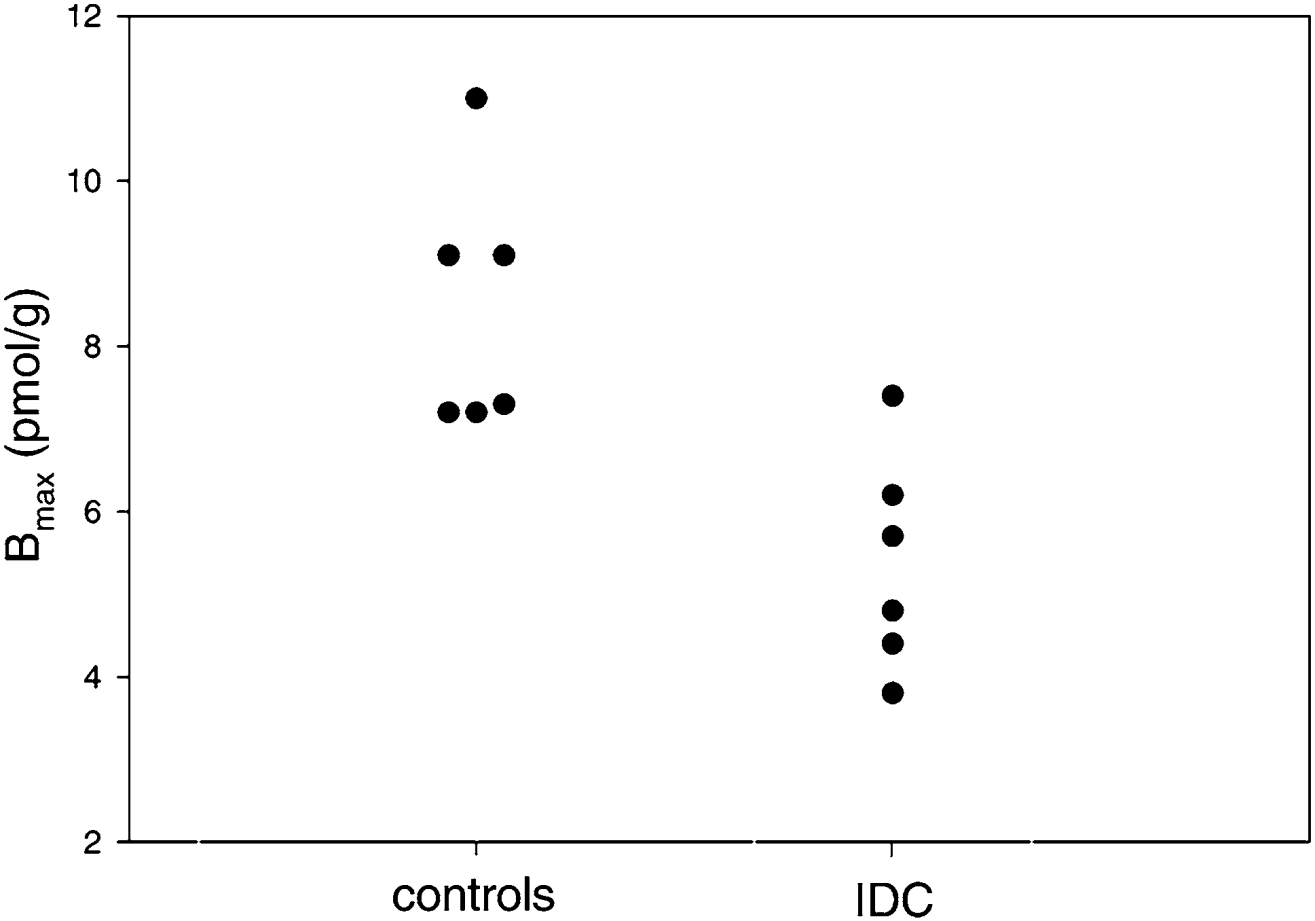
Graph of the individual β-AR densities as measured with PET for healthy controls (8.4 ± 1.5 pmol/g, *n* = 6) and patients with IDC (5.4 ± 1.3 pmol/g, *n* = 6). This research was originally published in Eur J Nucl Med Mol Imaging, re-used with permission [37]

Before PET scanning, patients have to withdraw short acting β-blockers. After injection of approximately 200 MBq of ^11^C-CGP, dynamic imaging will be performed, and heart rate and blood pressure will be monitored continuously. For kinetic data analysis of [^11^C]-CGP tracers, no ‘simplified’ method as the retention index is available (yet). Kinetic modelling of these postsynaptic PET tracers, and thus quantification of β-adrenoceptor density, is based on complex compartment models with either dual- or triple-injection protocols, consisting of different doses of high and low specific activity [17, 31]. In general, a first dose with a high specific activity will be followed 20 min later by a second dose with added non-radioactive ligand. A third injection consisting of non-radioactive ligand only is followed again 20 min later. Data acquisition starts at the onset of the first injection. Arterial blood samples are drawn during the first 20 min, and the radioactivity in the plasma is determined with a gamma counter. Radioactivity in the cellular fraction is calculated using plasma and whole blood data, in combination with the measured haematocrit value. The β-adrenoceptor density is eventually expressed as *B*_max_ in pmol/g myocardial tissue. Typically, β-adrenoceptor density is different between heart failure patients and healthy control subjects. For example, β-adrenoceptor density determined with [^11^C]-CGP-12388 was significantly lower in six idiopathic dilated cardiomyopathy patients compared to six age-matched healthy volunteers (Fig. 4).

### Relation pre- and postsynaptic sympathetic imaging: [^11^C]-CGP-12177 versus [^11^C]-mHED

**Fig. 5:**
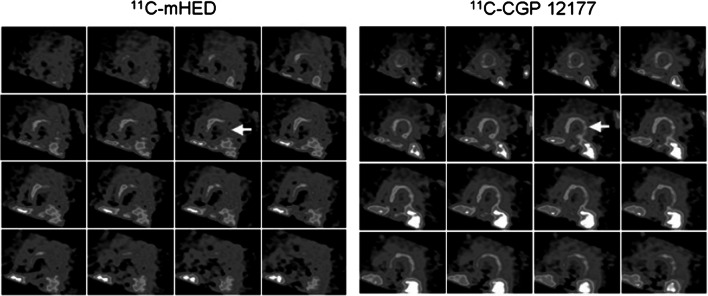
Short-axis PET images of [^11^C]-mHED (35- to 45-min sum) and [^11^C]-CGP (10- to 20-min sum from injection 1) in CHF patient. Apical slices are at upper left and basal slices are at lower right of each panel. Arrows indicate extensive mismatch between [^11^C]-mHED and [^11^C]-CGP. This research was originally published in JNM. Caldwell JH, Link JM, Levy WC, Poole JE, Stratton JR. Evidence for pre- to postsynaptic mismatch of the cardiac sympathetic nervous system in ischemic congestive heart failure. J Nucl Med. 2008;49:234–241. © by the Society of Nuclear Medicine and Molecular Imaging, Inc

At present, only a few studies have combined the acquisition of both the postsynaptic radiopharmaceutical [^11^C]-CGP-12177 and presynaptic radiopharmaceutical [^11^C]-mHED in the same patient cohort and healthy control subjects. In healthy controls, the distribution of both [^11^C]-CGP-12177 and [^11^C]-mHED is homogeneous and well matched [32]. In heart failure patients, both presynaptic and postsynaptic functions are described significantly different from healthy control subjects. In addition, a mismatch of [^11^C]-CGP-12177 versus [^11^C]-mHED uptake (Fig. 5) can be found in severe heart failure patients [14]. A (large) regional presynaptic defect with preserved postsynaptic β-adrenoceptor density is associated with worse outcome, for example death, cardiac arrest or progressive heart failure leading to transplantation. Also in patients with hibernating myocardium after infarction, a reduction in presynaptic uptake and postsynaptic β-adrenoceptor density was observed [33]. In these patients, the observed reduction was more global, in contrast to regional in patients with heart failure. Finally, in patients with arrhythmogenic right ventricular dysplasia/cardiomyopathy β-adrenoceptor downregulation, but no presynaptic innervation abnormality was reported [34]. This was suggested to be caused by regional elevated levels of catecholamines, leading to downregulation of β-adrenoceptors.

### Quantification software

At present, commercially available software for kinetic analysis of myocardial innervation PET tracers is rather limited. For that reason, many centers performing innervation PET studies have developed their own research software. Software packages originally introduced for the analysis and quantification of myocardial blood flow also allow processing of other types of dynamically acquired data, automatic segmentation of the myocardium and the subsequent generation of time–activity curves. This software enables regional analysis and replaces the concept of manually drawing VOIs. Such packages include FlowQuant^®^, Carismas, and HOQUTO [35].

## Future perspectives

Despite the strong properties of PET in general, non-invasive imaging of the autonomic innervation of the heart does not have a prominent role in clinical decision making thus far. However, the first large clinical trial (PAREPET) showed that assessment of regional myocardial denervation using [^11^C]-mHED predicts sudden cardiac death, independently of ejection fraction or infarct volume [4]. This can be considered as the step up to clinical implementation of this promising imaging modality.

PET tracers have the unique ability to identify sympathetic neurons by uptake and storage of tracers, and postsynaptic receptor binding affinity. Until recently, ligands for autonomic imaging had only labelled to radionuclides with a short half-life, making it only possible to perform imaging in those centers with an on-site cyclotron. The recent introduction of [^18^F] labelled tracers for sympathetic innervation will lead to the application of these tracers in non-cyclotron centers. At present, no β-adrenoceptor subtype-specific [^18^F] labelled tracer has been developed yet, and may be interesting for the near future. In addition, the rapid developments in gallium-68 and copper-64 radiochemistry could be of additional value for imaging of autonomic innervation in the future. However, this also requires further investigation. Imaging of β-adrenoceptors is complicated by application in a patient population that is frequently treated with chronic β-adrenoceptor blockade. This necessitates the development of novel tracers targeting intracellular second messengers and response elements of the signal cascade.

As stated before, dedicated software packages for dynamic data analysis are available, however, not specific for autonomic myocardial quantification. Due to the rapid developments in the field of innervation imaging, there is a growing need for easy-to-use software packages for reliable visual and quantitative interpretation.

Future developments in the field of autonomic innervation imaging should also focus on the cost-effectiveness of implantable cardioverter-defibrillators (ICDs) in patients with ischaemic cardiomyopathy. Since a minority of these patients will develop ventricular arrhythmia, the clinical need for better identification of patients who will benefit from ICD therapy is undisputable. Determination of the volume of denervated myocardial tissue using nuclear medicine modalities plays an additional role in better risk assessment of patients at risk for ventricular arrhythmia [4, 36]. Accordingly, imaging the extent of myocardial sympathetic denervation could be able to better identify those patients who will benefit from ICD therapy. With the help of healthcare insurance agencies, the reduction of costs from expensive ICDs can be achieved by imaging-based identification of those patients.

Finally, the developments in hybrid imaging leading to the simultaneous (or sequential) acquisition of PET and magnetic resonance imaging (PET/MRI) data can provide a further area of investigation with regard to tissue characterisation. The combination of a regional defect on sympathetic tracer imaging with either late gadolinium enhancement or abnormal non-contrast T1 mapping could potentially better identify the area of origin of ventricular arrhythmia.

## Conclusions

Non-invasive imaging of both pre- and postsynaptic sympathetic innervation using different PET tracers is still challenging and time consuming, due to complicated data analysis and lacking dedicated software. Experience is, up to present, mainly limited to centers with onsite cyclotrons. However, the increasing number of studies showing the additional value in cardiology, and the potential of a [^18^F] labelled tracer make sympathetic innervation imaging very attractive tool for further implementation in clinical decision making. To image β-adrenoceptors in cardiovascular patient population that is frequently treated with chronic β-adrenoceptor blockade, the development of novel tracers targeting intracellular second messengers and response elements of the signal cascade is needed.
